# Common variable immunodeficiency: an important but little-known risk factor for gastric cancer

**DOI:** 10.1590/0100-6991e-20213133

**Published:** 2021-12-01

**Authors:** PAULA KREIN, GUSTAVO GONÇALVES YOGOLARE, MARINA ALESSANDRA PEREIRA, OCTAVIO GRECCO, MYRTHES ANNA MARAGNA TOLEDO BARROS, ANDRE RONCON DIAS, ANA KAROLINA BARRETO BERSELLI MARINHO, BRUNO ZILBERSTEIN, CRISTINA MARIA KOKRON, ULYSSES RIBEIRO-JÚNIOR, JORGE KALIL, SERGIO CARLOS NAHAS, MARCUS FERNANDO KODAMA PERTILLE RAMOS

**Affiliations:** 1 - Faculdade de Medicina, Universidade de São Paulo, Curso de Medicina - São Paulo - SP - Brasil; 2 - Hospital das Clinicas HCFMUSP, Faculdade de Medicina, Universidade de São Paulo, Gastroenterologia - São Paulo - SP - Brasil; 3 - Hospital das Clinicas HCFMUSP, Faculdade de Medicina, Universidade de São Paulo, Clínica Médica - Disciplina de Imunologia Clínica e Alergia - São Paulo - SP - Brasil

**Keywords:** Stomach Neoplasms, Common Variable Immunodeficiency, Epidemiology, Risk Factors, Surgical Oncology, Neoplasias Gástricas, Imunodeficiência de Variável Comum, Epidemiologia, Fatores de Risco, Oncologia Cirúrgica

## Abstract

**Introduction:**

although it is a rare disease, common variable immunodeficiency (CVID) stands out as the most frequent primary symptomatic immunodeficiency. Carriers are prone to a variety of recurrent bacterial infections, in addition to the risk of developing autoimmune diseases and neoplasms including gastric cancer (GC). Despite the recognized risk, there are no specific standardized protocols for the management of GC in these patients, so the reported oncological results are varied. Thus, this study aims to describe the clinicopathological characteristics and prognosis of patients with CVID undergoing surgical treatment of GC.

**Methods:**

all patients with GC undergoing surgical treatment between 2009 and 2020 were retrospectively evaluated. Later, patients diagnosed with CVID were identified and this group was compared with the remaining patients without any immunodeficiency*.*

**Results:**

among the 1101 patients with GC evaluated in the period, 10 had some type of immunodeficiency, and 5 were diagnosed with CVID. Patients with CVID had younger age, lower BMI, and smaller lesions compared to those without CVID. Four patients underwent curative gastrectomy and one patient underwent jejunostomy. Two patients died (1 palliative and 1 curative) and one patient had disease recurrence. There was no statistically significant difference regarding the incidence of postoperative complications and survival between the evaluated groups.

**Conclusion:**

the CVID incidence in patients with GC undergoing surgical treatment was 0.5%, occurring at a less advanced age, but with no difference regarding surgical and oncological results.

## RESUMO

**Introdução:**

embora seja uma doença rara, a imunodeficiência comum variável (IDCV) destaca-se como a imunodeficiência primária sintomática mais frequente. Os portadores são propensos a uma série de infecções bacterianas recorrentes, além do risco de desenvolver doenças autoimunes e neoplasias incluindo o câncer gástrico (CG). Apesar do risco reconhecido, não existem protocolos específicos padronizados para o manejo do CG nesses pacientes, de modo que os resultados oncológicos relatados são variados. Assim, esse estudo tem como objetivo descrever as características clinicopatológicas e prognóstico de pacientes com IDCV submetidos ao tratamento cirúrgico do CG.

**Métodos:**

foram avaliados retrospectivamente todos os pacientes com CG submetidos a tratamento cirúrgico entre 2009 e 2020. Posteriormente foram identificados pacientes com diagnóstico de IDCV e esse grupo foi comparado com o restante dos pacientes sem nenhuma imunodeficiência.

**Resultados:**

dentre os 1101 pacientes com CG avaliados no período, 10 apresentavam algum tipo de imunodeficiência e 5 foram diagnosticados com IDCV. Os pacientes com IDCV apresentaram idade menos avançada, menor IMC e lesões com menor diâmetro em comparação aqueles sem IDCV. Quatro pacientes foram submetidos à gastrectomia curativa e um paciente realizou jejunostomia. Dois pacientes foram a óbito (1 paliativo e 1 curativo) e um paciente apresentou recidiva da doença. Não houve diferença estatisticamente significativa em relação à incidência de complicações pós-operatórias e sobrevida entre os grupos avaliados.

**Conclusão:**

a incidência IDCV nos pacientes com CG submetidos à tratamento cirúrgico foi de 0,5% ocorrendo em idade menos avançada mas sem diferença com relação aos resultados cirúrgicos e oncológicos.

## INTRODUCTION

Gastric cancer (GC) is a globally distributed disease, being the fifth most common cancer and the third leading cause of cancer-related mortality, with more than 1,033,701 new cases and about 780,000 deaths per year worldwide^1^. In Brazil, it is estimated that there were 21,290 new cases of gastric cancer in 2018, being the fourth tumor with the highest incidence in males and the sixth in females^2^.

Risk factors commonly related to the development of gastric cancer include chronic infection with Helicobacter pylori (H. pylori), low fruit and vegetable intake, high salt intake, smoking, and alcohol consumption^3^ ^-^ ^5^. The World Health Organization (WHO) classifies H. pylori as a group 1 carcinogen for humans. It is one of the most common infections in the world, with an estimated prevalence of 50%, reaching 90% in developing countries. However, only a small portion of individuals infected with H. pylori develops GC, presupposing interaction with environmental factors - such as smoking and alcohol consumption - in individuals with genetic susceptibility, in addition to the variation in the bacterial strain^6^ ^,^ ^7^. 

Another known risk factor, though rarely mentioned, is the presence of Primary Immunodeficiencies (PID), which, in addition to increasing the risk of developing GC, causes its manifestation at earlier ages than in the general population^8^. PIDs are a set of diseases that comprise more than 300 innate immunity defects, the majority of which of unknown cause. People with PID are at increased risk of recurrent and chronic infections, autoimmune diseases, and cancer throughout life. Followed by infections, the occurrence of neoplasms is the second most common cause of death in this population. Between 4% and 25% of patients with PID will develop some neoplasm^9^. Specifically, the risk of developing GC is around three to four times higher in this population^10^. 

Among PIDs, Common Variable Immunodeficiency (CVID) is one of the most associated with antibody deficiency. Patients with CVID have an even greater risk of developing GC, which can be up to 10 times higher than the general population^11^. Patients with CVID have a higher incidence of premalignant lesions (atrophic gastritis, intestinal metaplasia, and dysplasia) when compared with the general population, with a faster evolution to malignant lesions^12^. However, despite the higher risk of neoplastic development attributed to these patients, there are no specific standardized protocols for the treatment of GC in patients with CVID, so the reported oncological results vary according to the different populations analyzed.

Thus, this study aims to describe the clinicopathological characteristics of patients with GC and CVID undergoing surgical treatment, along with the surgical and oncological results. 

## METHODS

We retrospectively evaluated all patients with GC undergoing surgical treatment between 2009 and 2020. We gathered the data from the Service’s Database, which is maintained prospectively. Patients with a histological diagnosis of gastric adenocarcinoma were eligible for the study. The diagnosis of immunodeficiency was established at the Ambulatory of Immunodeficiencies, Service of Immunology, Department of Internal Medicine. We excluded patients with acquired immunodeficiencies and other types of congenital immunodeficiencies other than CVID. As a comparison group, we used patients with gastric adenocarcinoma undergoing surgical treatment in the same period, without any immunodeficiency.

Sex, age, body mass index (BMI), hemoglobin level, albumin level, neutrophil-lymphocyte ratio (NLR), American Society of Anesthesiology (ASA) classification, and presence of comorbidities according to the Charlson-Deyo classification^13^ comprised the studied clinical features. We assessed postoperative complications using the Clavien-Dindo classification^14^, with major postoperative complications being defined as Clavien-Dindo grades III to V.

Preoperative staging was performed using computed tomography of the abdomen and pelvis, endoscopy, and laboratory tests. The patients were staged according to the TNM, 8^th^ edition. All patients were operated on by experienced surgeons and in accordance with current recommendations^15^ ^,^ ^16^. 

Postoperative follow-up occurred quarterly in the first year and every six months in the following years. Follow-up examinations to detect recurrence were performed based on the presence of symptoms. Absence from appointments for more than 12 months was considered as loss to follow-up. The present study was approved by the Institution’s Ethics in Research Committee (Plataforma Brasil, CAAE:39341920200000068).

### Statistical analysis

Descriptive statistics included frequency analysis, with percentage for nominal variables, and means with standard deviation for continuous variables. We used the t test and the Fisher’s exact test to assess continuous and categorical variables, respectively. We estimated disease-free survival (DFS) and overall survival (OS) with the Kaplan-Meier method and examined differences between the curves using the Log-Rank test. We calculated survival time from the date of operation until the date of death or disease recurrence. Living patients were censored on the date of the last visit. We considered values of p<0.05 as statistically significant. We performed the analyzes using the SPSS software, version 20.0 (SPSS Inc, Chicago, IL).

## RESULTS

Among the 1,101 patients undergoing surgical treatment by GC in the referenced period, 10 (0.9%) had some immunodeficiency. Of these, five patients had CVID and were included in the study. Among the cases excluded due to the presence of another immunodeficiency, two had acquired immunodeficiency syndrome (AIDS), two had monoclonal gammopathy, and one patient had no defined immunodeficiency diagnosis.

All patients with CVID had regular endoscopies as part of a screening program, four of them annually and one biannually. Four patients had previous gastric biopsy with intestinal metaplasia. The search for H. pylori was negative in all cases. Regarding symptoms, four patients reported chronic diarrhea.

The clinical characteristics of the five patients with CVID in relation to all patients with GC without immunodeficiencies treated in the period are shown in Table 1. Patients with CVID were younger (50.8 years vs. 62.9 years, p=0.030) and had lower BMI (18.5 vs. 23.7 kg/m^2^, p=0.019) compared with those without CVID.

**Table 1:** Clinical characteristics of patients with gastric adenocarcinoma with and without CVID.

Variables	GC without CVID n=1096	CVID n=5 (%)	p-value
Sex			1
Female	409 (37.3)	2 (40)	
Male	687 (62.7)	3 (60)	
Age (years)			0.03
Mean (SD)	62.9 (12.7)	50.8 (10.9)	
Body mass index (kg/m²)			0.019
Mean (SD)	23.7 (4.9)	18.5 (1.7)	
Hemoglobin (g/dL)			0.44
Mean (SD)	11.8 (4.4)	13.3 (1.25)	
Albumin (g/dL)			0.617
Mean (SD)	3.86 (1.15)	4.12 (0.6)	
Neutrophil-lymphocyte ratio (NLR)			0.691
Mean (SD)	3.48 (3.97)	2.77 (1.27)	
Charlson-Deyo Comorbidity Index			0.658
0	745 (68)	3 (60)	
≥1	351 (32)	2 (40)	
ASA (American Society of Anesthesiologists) classification			0.656
I / II	748 (68.2)	3 (60)	
III / IV	348 (31.8)	2 (40)	
cTNM			0.656
I / II	467 (42.6)	3 (60)	
III / IV	629 (57.4)	2 (40)	

Among the five patients with CVID, four underwent gastrectomy with curative intent, and one, palliative jejunostomy due to the presence of liver metastases. Table 2 shows the pathological characteristics of patients with and without CVID undergoing curative treatment. Patients with CVID had smaller diameter tumors compared with the group without this condition (1.4cm vs. 4.7cm; p=0.025).

**Table 2:** Pathological characteristics of patients with CVID undergoing gastric resection.

Variables	GC without CVID n=596 (%)	CVID n=4 (%)	p-value
Tumor size			0.025
Mean (SD)	4.7 (2.9)	1.4 (0.94)	
Type of gastrectomy			1
Subtotal	386 (64.8)	3 (75)	
Total	210 (35.2)	1 (25)	
Lymphadenectomy			0.61
D1	102 (17.1)	0 (0)	
D2	494 (82.9)	4 (100)	
Lauren's histological type			0.63
Intestinal	325 (54.5)	3 (75)	
Diffuse/Mixed	271 (45.5)	1 (25)	
Differentiation			0.05
G1/G2	281 (47.1)	4 (100)	
G3	315 (52.9)	0 (0)	
Lymphatic invasion			0.624
No	306 (51.3)	3 (75)	
Yes	290 (48.7)	1 (25)	
Venous invasion			0.309
No	399 (66.9)	4 (100)	
Yes	197 (33.1)	0 (0)	
Perineural invasion			0.627
No	316 (53)	3 (75)	
Yes	280 (47)	1 (25)	
pT			0.315
pT1/T2	249 (41.8)	3 (75)	
pT3/T4	347 (58.2)	1 (25)	
Number of LN dissected			0.7
Mean (SD)	40.7 (18.1)	37.3 (19.4)	
LNM			0.324
pN0	260 (43.6)	3 (75)	
pN+	336 (56.4)	1 (25)	
pTNM			0.637
I / II	337 (56.5)	3 (75)	
III / IV	259 (43.5)	1 (25)	

The mean length of hospital stay of patients was 8.3 days and 12.1 days in the CVID and non-CVID groups, respectively (p=0.417). No patient with CVID had major postoperative complications. Likewise, there was no postoperative mortality at 30 and 90 days (Table 3).

**Table 3:** Surgical and oncological results of patients with CVID undergoing gastric resection.

Variables	GC without CVID n=596 (%)	CVID n=4 (%)	p-value
Length of stay (days)			0.417
Mean (SD)	12.1 (9.5)	8.3 (2.4)	
Grade of postoperative complication			0.642
0 - I - II	512 (85.9)	4 (100)	
III - IV - V	84 (14.1)	0 (0)	
30-day mortality			1
No	575 (96.5)	4 (100)	
Yes	21 (3.5)	0 (0)	
90-day mortality			1
No	553 (92.8)	4 (100)	
Yes	43 (7.2)	0 (0)	
Adjuvant Chemotherapy			0.054
No	286 (48)	4 (100)	
Yes	310 (52)	0 (0)	
Survival			
DFS (%)	71.40%	75%	0.733*
SG (%)	56.70%	75%	0.795*

DFS: disease-free survival; SG: overall survival.*Log Rank Test

During the follow-up period, of the four patients treated with curative intent, one died without evidence of disease and one relapsed. The patient submitted to jejunostomy died 12.3 months after the operation. 

Regarding the survival of patients undergoing potentially curative surgery, the DFS rate was 56.7% and 75.0% for the group without CVID and with CVID, respectively (p=0.795). OS was similar between groups, 71.4% in patients without CVID and 75.0% in those with CVID (p=0.733).

Table 4 summarizes the characteristics of each of the five patients with CVID analyzed in the study.

**Table 4:** Summary of characteristics of patients with CVID included in the study.

Case	Age	Sex	BMI	Hb	Alb	H.pylori	AG*	IM*	Diarrhea	Surgery	Location	Lauren	Size	pTNM	EC	DFS	SG
1	63	F	16.9	11.8	3.2	Negative	No	No	Yes	Jejunostomy	Body	-	-	-	IVB	0.0	12.3
2	51	F	20.0	13.9	4.6	Negative	No	Yes	Yes	Subtotal D2	Body	Intestinal	0.2	T1a N0 M0	IA	55.6	55.6
3	42	M	19.4	14.3	3.8	Negative	No	Yes	Yes	Subtotal D2	Antrum	Intestinal	2.5	T2 N0 M0	IB	47.8	47.8
4	38	M	16.6	12.1	4.3	Negative	No	Yes	Yes	Subtotal D2	Antrum	Mixed	1.4	T1a N0 M0	IA	7.1	7.1
5	60	M	19.8	14.4	4.7	Negative	No	Yes	No	Total D2	Antrum	Intestinal	1.5	T4a N3a M0	IIIC	4.1	14.6

*AG: atrophic gastritis; IM: intestinal metaplasia.

## DISCUSSION

We evaluated a series of cases of patients with GC who presented CVID and underwent surgical treatment in a single reference center. We found that tumors in patients with CVID occurred at an earlier age. However, there were no differences in pathological and survival outcomes between the groups. Surgical complications, which are a major concern in immunocompromised patients, were also not different between the evaluated groups.

In patients with PIDs, the presence of gastrointestinal disorders is quite frequent, occurring between 5% and 50% of cases. This is in part because the intestine is the largest lymphoid organ in the human body, containing most lymphocytes and producing large amounts of immunoglobulins. Gastrointestinal manifestations can be related to infection, inflammation, autoimmune diseases, and neoplasms^17^. When compared with the same age group, patients with CVID are at increased risk of developing cancer, particularly non-Hodgkin’s lymphomas, leukemia, and gastric cancer^18^. 

Common variable immunodeficiency (CVID) is the most common form of PID, and the prevalence is estimated at 1 in 25,000 to 50,000 people. Its pathogenesis has not been fully elucidated, however mutations of several genes related to the development of B cells in immunoglobulin-producing plasma cells and memory B cells have been described^19^. Affected individuals commonly present with recurrent upper and lower respiratory tract bacterial infections, autoimmune diseases, granulomatous infiltrative disease, and neoplasms. The most common tumors are lymphoma, gastric cancer, and breast cancer^20^. Diagnosis is based on the significant reduction in serum levels of IgG, IgA, and/or IgM, in addition to reduced production of antibodies after the application of vaccines. Most patients are diagnosed between the ages of 20 and 40, and treatment consists of monthly administration of immunoglobulin^19^. 

The increased risk of GC in patients with CVID varies according to the incidence rate of GC in patients without CVID in the evaluated country. A Scandinavian study estimated an increased risk of 10 times^11^, while an Australian one showed an increased risk of 7.23 times^18^. Although there is no conclusive evidence, the most accepted mechanism for the increased risk of GC in the presence of CVID is due to the reduction in gastric IgA and hydrochloric acid production - factors that promote chronic gastritis and facilitate colonization by H. pylori, triggering carcinogenesis. This mechanism is supported by the finding that patients with pernicious anemia, who also have achlorhydria and chronic gastritis, have a three times greater risk of developing GC19. The decrease in local immune response also may play a role in neoplastic development, due to the lower number of B cells in the gastric mucosa of the patients with CVID.

Cancer diagnosis in patients with CVID usually occurs at an earlier age, on average 15 years earlier than in the general population, a characteristic that was also verified in our study. Regarding the histological diagnosis of the tumor, Lauren’s Intestinal type is usually the most frequent, being moderately or poorly differentiated and containing a high number of intra-tumoral lymphocytes. In addition, atrophic pangastritis with little presence of plasma cells, nodular lymphoid aggregates, and apoptotic activity are usually present due to the associated autoimmune gastritis^19^. 

Given the evidence of increased risk of developing GC, it is important that patients with CVID are included in early screening programs^12^ ^,^ ^19^ ^,^ ^21^. Dutch data have shown a high incidence of premalignant histological and/or endoscopic lesions in patients with CVID, such as atrophic gastritis, intestinal metaplasia, and dysplasia, even in asymptomatic individuals. Up to 88% of patients with CVID with no prior gastrointestinal history may have premalignant lesions on endoscopy. The progression rates of these lesions to GC vary between 0% and 1.8% per year in atrophic gastritis, from 0% to 10% per year for intestinal metaplasia, and between 0% and 73% per year when dysplasia is already present^12^. 

It is recommended that patients with chronic atrophic gastritis undergo endoscopy every three years, while for those with intestinal metaplasia the exam can be indicated every one to three years, reducing to a frequency of six to 12 months in cases of dysplasia^21^. However, these intervals may not be appropriate for patients with CVID, as GC development may occur more quickly. In fact, there is no standardized screening protocol, and the indication must consider the regional GC incidence. 

Patients with CVID can develop neoplasia 12 to 14 months after an endoscopy without signs of dysplasia. This justifies the proposal to indicate endoscopy for all patients with CVID at the time of diagnosis, repeat it every 24 months in patients with normal histology, every 12 months in patients with atrophic gastritis or intestinal metaplasia, and every six months in patients with dysplasia. Routine eradication of H. pylori is also recommended^19^ ^,^ ^21^. In the present study, all included patients underwent endoscopies regularly and did not display H. pylori infection. Thus, the effectiveness of screening can be evidenced in part by the smaller size of tumors in the CVID group, although we observed no differences in terms of staging and survival between groups, which may also be due to the small number of patients evaluated with CVID. 

There are no specific protocols for the treatment of cancer in patients with CVID. Once the GC diagnosis is made, these patients must undergo standard treatment, the same offered to the immunocompetent population^19^ ^,^ ^22^. Preoperative nutritional therapy and administration of immunoglobulin are recommended measures. CVID patients can receive the same chemotherapy protocols used in immunocompetent patients^23^. However, short-term protocols are preferable to long-term regimens, with special attention to infection control and prophylaxis for Pneumocystis jirovencii pneumonia^22^ ^,^ ^23^. When possible, the chemotherapy regimen should be tailored according to individual risk factors and tolerance^23^. 

The present study has some limitations, especially the small number of patients with CVID, a fact that limits some analyzes and makes the results more descriptive. However, even with small samples, highlighting characteristics and aspects related to patients with GC who have CVID is important to increase knowledge about this type of immunodeficiency as a risk factor for GC, in addition to reinforcing the importance of adopting rigorous screening protocols in such patients.

## CONCLUSION

The incidence of Common Variable Immunodeficiency in patients with gastric cancer undergoing surgical treatment was 0.5%. Patients with CVID were diagnosed with GC at a younger age, on average 12 years before the control population. Tumor stage, surgical outcomes, and survival were similar between groups. Thus, we recommend that patients with CVID be promptly included in screening programs to detect GC in early stages.
